# Microwave-Assisted Classic Ullmann C–C Coupling Polymerization for Acceptor-Acceptor Homopolymers

**DOI:** 10.3390/polym11111741

**Published:** 2019-10-24

**Authors:** Zijie Li, Yusheng Chen, Pan Ye, Xiangli Jia, Xiaoxi Wu, Jianfei Wu, Qinqin Shi, Aidong Peng, Hui Huang

**Affiliations:** College of Materials Science and Opto-Electronic Technology & Center of Materials Science and Optoelectronics Engineering & CAS Center for Excellence in Topological Quantum Computation & CAS Key Laboratory of Vacuum Physic, University of Chinese Academy of Sciences, Beijing 100049, China; lizijie_win@163.com (Z.L.); chenyusheng767@gmail.com (Y.C.); yepan1108@163.com (P.Y.); jiaxiangli14@mails.ucas.edu.cn (X.J.); estherwuxiaoxi@gmail.com (X.W.); wujianfeidbj@gmail.com (J.W.); paidong@ucas.ac.cn (A.P.)

**Keywords:** A-A homopolymers, classic Ullmann coupling polymerization, microwave-assistance, organic solar cells

## Abstract

Developing cheap, clean and atomic-efficient synthetic methodologies for conjugated polymers are always critical for the field of organic electronics. Herein, classic Ullmann coupling polymerization is developed to synthesize a series of Acceptor-Acceptor (A-A) type homopolymers with microwave-assistance, which are supported by nuclear magnetic resonance (NMR), matrix-assisted laser desorption/ionization time of flight mass spectrometry (MALDI-TOF), elemental analysis (EA) and gel permeation chromatography (GPC). The physicochemical properties of these polymers are studied by UV-vis spectroscopy, cyclic voltammetry (CV), thermal gravimetric analysis (TGA), and density functional theory (DFT) calculation. Furthermore, these A-A homopolymers are used as acceptors for all-polymer solar cells (All-PSCs), affording a promising efficiency of 3.08%, which is the highest value for A-A-homopolymer-based organic solar cells.

## 1. Introduction

Polymeric semiconductors are emerging as crucial components for state-of-the-art opto-electronic technologies, including organic thin film transistors (TFTs), organic photovoltaics (OPV) and organic photodetectors (OPDs), due to their tunable energy levels, potentially low cost manufacturing, and solution-processability [1,2,3,4]. To tune the energy levels and enhance intramolecular charge transfer, the donor-acceptor (D-A) strategy has been used to construct conjugated polymers by various transition metal-mediated aryl coupling methodologies, including Stille coupling and Suzuki coupling reactions [5,6,7,8,9,10,11,12]. Although D-A type copolymers have demonstrated great success in the area of organic electronics, they still suffered from complicated structures and costly synthetic routes. In comparison, homopolymers demonstrated a promising future for commercialization due to their simple structures, easily synthetic routes, and potentially low-cost manufacture [13]. For example, poly(3-hexylthiophene) (P3HT), as one of the Donor-Donor (D-D) conjugated polymers available in quantities over 10 kg, was considered as a promising material for commercial electro-devices [14]. In addition, D-D homopolymer poly-4,8-bis-(5-((2-ethylhexyl)thio)thiophen-2-yl)benzo[1,2-b:4,5-b′]-dithiophene (PBDTT-S) based on benzo[1,2-b:4,5-b′]dithiophene (BDT) units was reported for OPVs with a high power conversion efficiency (PCE) of 7.05% [13]. On the other hand, acceptor-acceptor (A-A) homopolymers are relatively rare, although they exhibit excellent electronic affinities and air-stability for high-performance organic electronics [15,16,17,18,19]. For example, Guo and coworkers synthesized an A-A homopolymer, poly(2,2′-bithiazolothienyl-4,4′,10,10′-tetracarboxydiimide) (PDTzTI) through Stille coupling for organic field effect transistors (OFETs) devices, which exhibits a remarkable electron mobility (μ_e_) of 1.61 cm^2^ V^−1^ s^−1^ [20]. Reynolds et al. employed an isoindigo unit to synthesize poly(isoindigo) through Suzuki polycondensation, which was used as an acceptor for OPVs, affording a relatively low efficiency of 0.47% [21]. Unfortunately, these synthetic methods usually involve several synthetic steps and toxic reagents (e.g., tin), which is detrimental for commercializing organic electronic technologies. Thus, it is critical to develop cheap, clean and atomic-efficient synthetic methodologies for high-performance A-A homopolymers.

The classic Ullmann coupling reaction is a copper-mediated methodology used to build conjugated systems upon coupling of two molecules of aryl halides with C–C bond formation; its advantages include cheap reagents, straightforward routes, and environmentally friendly oxidants (air or oxygen) [22,23,24]. Thus, Ullmann coupling has been developed to construct molecular and polymeric conjugated materials for opto-electronics [25,26], medicines [27,28], and dyes [29]. For example, Gorgun et al. [25,26] employed Ullmann coupling to synthesize carbazole-based organic semiconductors for photodiodes and photodetectors with respectable performances. Wang and coworkers [27] used CuI as a catalyst to promote a domino reaction, where the condensation and cyclization reaction were followed by an intramolecular Ullmann type reaction, to give 16H-dibenzo[2,3:6,7][1,4]oxazepino[5,4-b]quinazolin-16-ones in good yields. However, the Ullmann coupling reaction has never been employed to synthesize A-A homopolymers.

In this contribution, classic Ullmann polycondensation was employed to synthesize three A-A homopolymers (PPDI(H), PPDI(OD), and PNDI(OD)) with microwave-assistance, which were supported by nuclear magnetic resonance (NMR), elemental analysis (EA), and gel permeation chromatography (GPC). The physicochemical properties of these polymers were investigated by UV-vis spectroscopy, cyclic voltammetry (CV), thermal gravimetric analysis (TGA), and density functional theory (DFT) calculations. Furthermore, these A-A polymers were employed as acceptors for all polymer solar cells (All-PSCs), affording a promising efficiency of 3.08%, which is the highest value for A-A homopolymer-based organic solar cells (OSCs).

## 2. Results and Discussion

### 2.1. Ullmann Polymerization

**Scheme 1:**
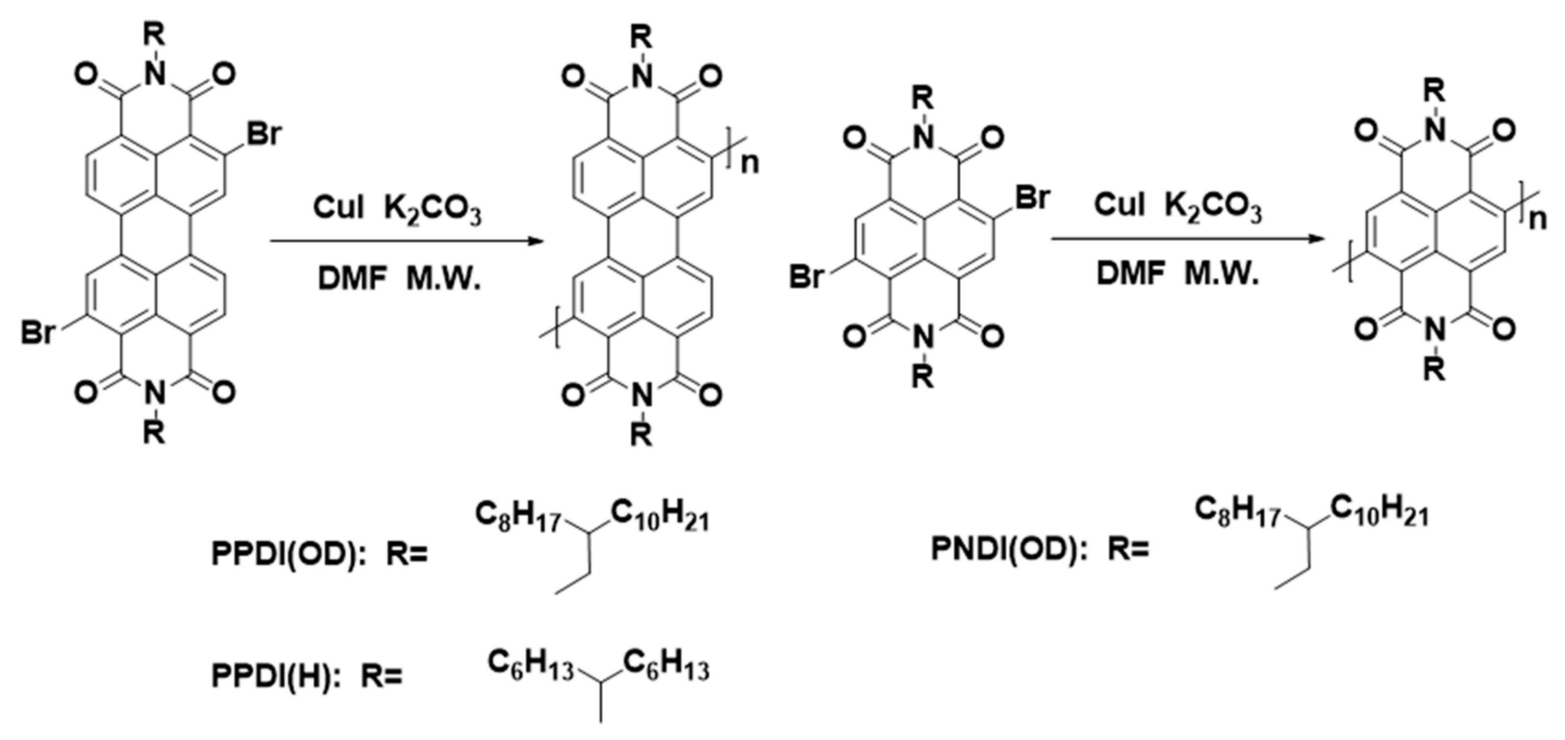
Synthetic routes of A-A homopolymers.

Scheme 1 shows the synthetic route of poly[5,12-dimethyl-2,9-di(tridecan-7-yl)anthra[2,1,9-def:6,5,10-d′e′f′]diisoquinoline-1,3,8,10(2H,9H)-tetraone] (PPDI(H)). The reaction conditions of Ullmann polymerization are listed in Table 1. In a typical reaction, PDI(H) was heated with 40% equiv. of cuprous iodide and 2 equiv. of base in a solvent. According to the literature [30], polar solvents are good choices for Ullmann polymerization in most instances. Thus, dimethyl sulfoxide (DMSO) was first chosen as the solvent for the polymerization at 80 °C (Entry 1). However, no product was obtained due to the poor solubility of 2Br-PDI in DMSO. Thus, toluene, another polar and excellent solvent for 2Br-PDI, [31] was used for the reaction (Entry 2), but no product was obtained at 80 °C. Considering temperature is a key parameter for catalytic reaction, when the temperature was raised to 150 °C, a good amount of oligomers with up to 5 repeating units were achieved after 12 h, confirmed by matrix-assisted laser desorption ionization time-of-flight (MALDI-TOF) (Entry 4, Figure S1). Afterwards, the reaction time was extended to 24 h to afford a higher yield and molecular weight (Entry 5, Figure S2). However, the molecular weight is still too low to be defined as a polymer.

The microwave-assistance technique has been used as an important tool for coupling polymerization [32,33]. Thus, this technique was employed to improve the efficiency of this Ullmann polymerization. Impressively, the repeating units were significantly improved to 11 units under microwave assistance (Entry 6, Figure S3), while the reaction time was dramatically reduced to 5 h. The number-average molecular weight (Mn) and polydispersity (PDI) are 7.4 kDa and 1.86, respectively, indicating that a polymeric material was achieved.

In order to investigate the versatility of the Ullmann polymerization methodology for A-A homopolymers, another two polymers PPDI(OD) and PNDI(OD) were successfully synthesized under the optimized reaction conditions. All three polymers were fully characterized by ^1^H-NMR, elementary analysis and GPC (Supporting Information, Figures S4–S9). According to the GPC results, the Mn and PDI of PPDI(OD) and PNDI(OD) are 12.8 kDa and 1.84, and 5.9 kDa and 1.86, respectively. These respectable Mn and PDI values suggest that versatile applications exist for the use of Ullmann coupling polymerization for synthesizing A-A conjugated polymers. Moreover, these three polymers are soluble in common solvents such as chloroform (CF), chlorobenzene (CB), tetrahydrofuran (THF) and hexane at room temperature. Thermal properties of the polymers were investigated by TGA. As shown in Figure S10, the onset temperatures at 5% weight loss for these polymers are over 280 °C, which indicated the excellent thermal stability. Moreover, differential scanning calorimetry (DSC) measurement (Figure S11) shows that all these polymers exhibit no obvious melting transition temperature, which indicated the relatively poor crystallinity due to the twisted conformation.

### 2.2. Optical and Electrochemical Properties

**Figure 1:**
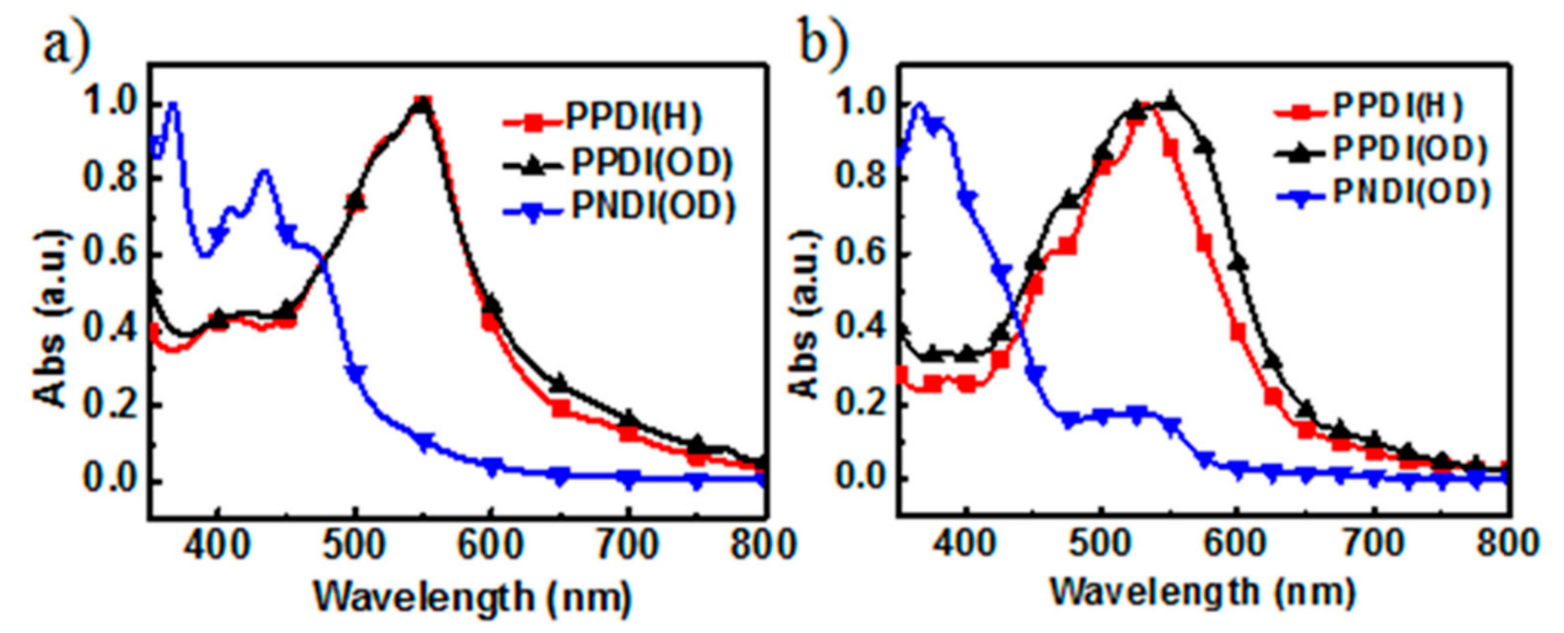
Normalized UV–vis spectra of these polymers in solution (**a**) and as film (**b**).

UV-vis optical absorption spectra and CV spectrum were performed to investigate the energy levels of PPDI(H), PPDI(OD) and PNDI(OD). The spectra are shown in Figure 1 and Figure S12, respectively and the data are listed in Table 2. In solution, both PPDI(H) and PPDI(OD) showed broad absorption bands with maximum absorption peaks at 549 nm and 546 nm, respectively. The similar absorption profiles indicate turning alkyl chain barely affect the optical properties. Unlike PDI-based homopolymers, the PNDI(OD) showed two absorption bands. The smaller one corresponds to the π–π* transition, while the larger peak is ascribed to the intramolecular charge transfer (ICT), suggesting the weak ICT process in PNDI(OD). In thin films, PPDI(OD) shows a maximum absorption peak at 545 nm, which is slightly red shifted (13 nm) in comparison with that PPDI(H), indicating the stereo-demanding alkyl chain may influence the intermolecular packing and thus the optical properties. Also, PNDI(OD) demonstrates two absorption peaks at 368 nm and 531 nm in film. Notably, these values are close to those of solutions, suggesting very weak intermolecular interactions. According to the onset of the long wavelength absorption, the optical band gaps (*E*_g_) of PPDI(H) and PPDI(OD) and PNDI(OD) were estimated to be 1.94 eV, 1.87 eV and 2.16 eV, respectively. According to the CV measurement, the LUMO energy levels of PPDI(H) and PPDI(OD) and PNDI(OD) were estimated to be −4.00 eV, −4.05 eV and −3.84 eV, respectively. Thus, HOMO energy levels of PPDI(H) and PPDI(OD) and PNDI(OD) were calculated to be −5.94 eV, −5.92 eV and −6.00 eV, respectively.

### 2.3. DFT Calculation

**Figure 2:**
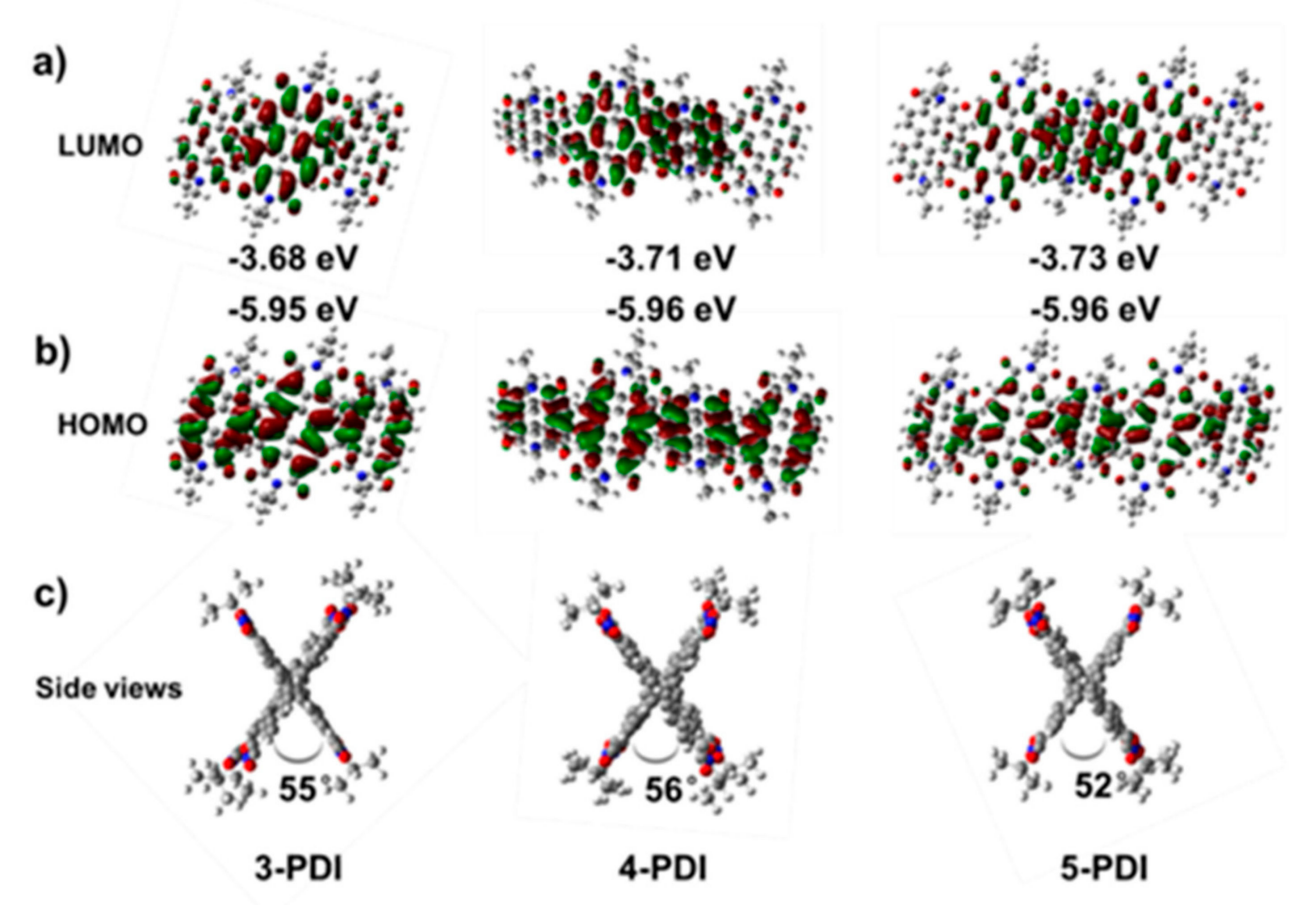
(**a**,**b**) Optimized geometry, calculated HOMO and LUMO orbitals of the 3-PDI, 4-PDI and 5-PDI; (**c**) optimized geometry of three molecules shown in side view.

In order to further understand the optical properties of PPDI and PNDI, DFT calculations were carried out at the level of the B3LYP/6-31G(d, p) as shown in Figure 2 and Figure S13, respectively. From the side view (Figure 2c), dihedral angles of these structures 3-PDI, 4-PDI, and 5-PDI are 55°, 56°, and 52° respectively, indicating the X-type structure of PPDI. Obviously, twisted molecules exhibit weak intermolecular interactions and prevent the strong aggregation and large domain size, which may be beneficial for optimal phase separation in the blend films of OSCs [34,35,36,37]. The molecular orbital distribution showed that the highest occupied molecular orbital (HOMO) is delocalized along the whole backbone of the molecules, while the lowest unoccupied molecular orbital (LUMO) is localized on the center part of these molecules, indicating moderate intramolecular charge transfer, consistent with the optical absorption. The DFT calculations (Table S2) showed that the energy levels of LUMO/HOMO of these three molecules (3-PDI, 4-PDI, and 5-PDI) are −3.68/−5.95 eV, −3.71/−5.96 eV, −3.73/−5.96 eV, respectively. Obviously, as the number of repeating units (PDI) increased, energy level of LUMO decreases but HOMO remains unchanged, which thus can narrow the bandgap.

### 2.4. Fabrication and Characterization of All-PSCs

Among these three A-A homopolymers, PPDI(H) exhibits complementary absorptions with narrow bandgap materials, such as poly[4,8-bis(5-(2-ethylhexyl)thiophen-2-yl)benzo[1,2-b:4,5-b′]dithiophene-co-3-fluorothieno[3,4-b]thiophene-2-carboxylate] (PTB7-Th) and poly[(2,6-(4,8-bis(5-(2-ethylhexyl)thiophen-2-yl)benzo[1,2-b:4,5-b′]dithiophene)-co-(1,3-di(5-thiophene-2-yl)-5,7-bis(2-ethylhexyl)benzo[1,2-c:4,5-c′]dithiophene-4,8-dione)] (PBDB-T) [38,39]. Furthermore, PPDI(H) presents a similar absorption coefficient to these two D-A donor conjugated polymers (Figure S14), suggesting that PPDI(H) may be an excellent acceptor matching with these two donors for All-PSCs.

**Figure 3:**
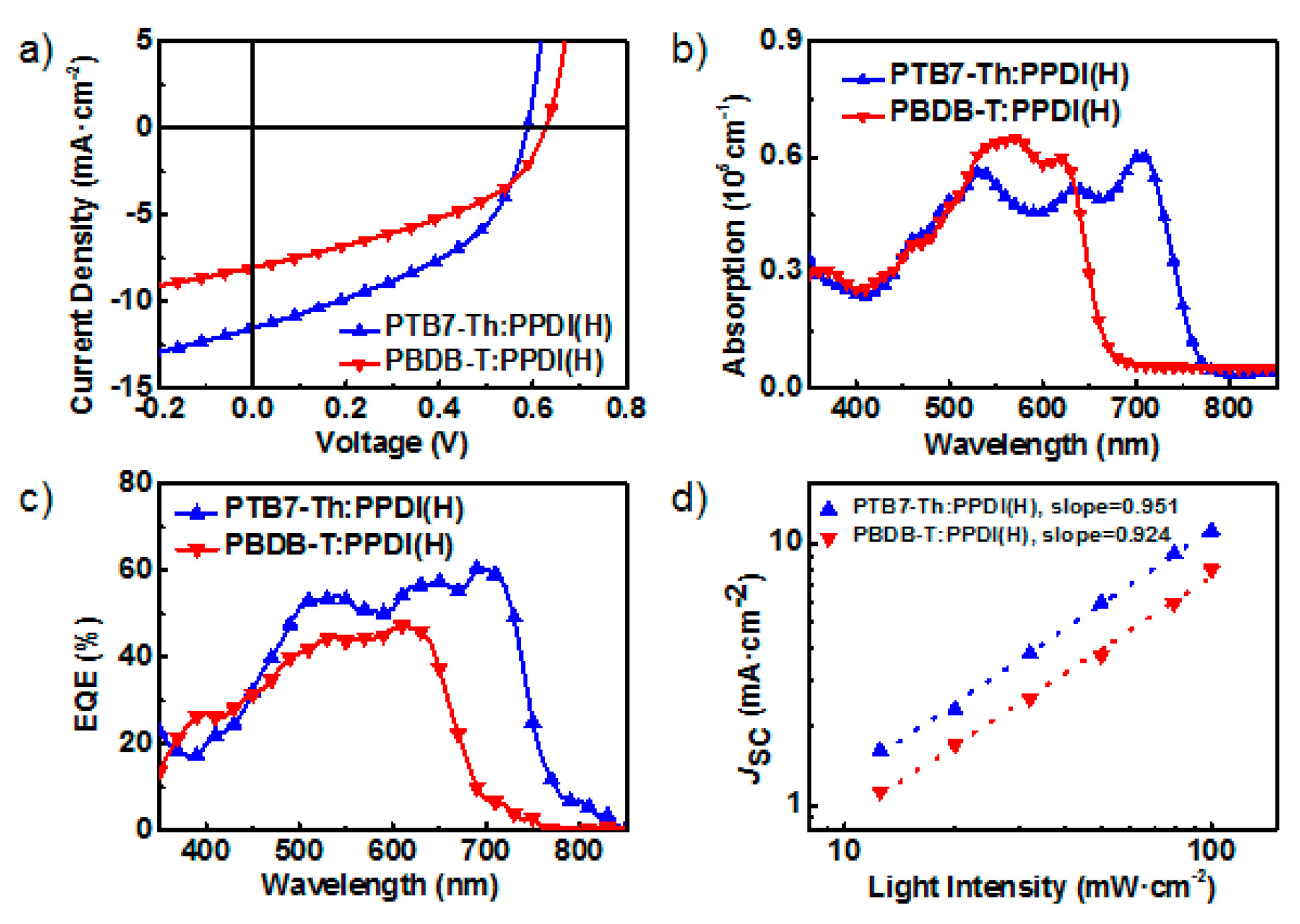
(**a**) The current density−voltage (J-V) curves of PPDI(H)-based All-PSCs; (**b**) absorption coefficient of PTB7-Th:PPDI(H) and PBDB-T:PPDI(H) blend films; (**c**) EQE of PPDI(H)-based All-PSCs; (**d**) short current density (*J_SC_*) versus light intensity of the PTB7-Th-based devices and PBDB-T-based devices.

To investigate the photovoltaic performances of PPDI(H)\PTB7-Th:PPDI(H) and PBDB-T:PPDI(H) based All-PSCs were fabricated with inverted structures of ITO/ZnO/Active layer/MoO3/Ag. The typical current density−voltage (J-V) curves are shown in Figure 3a and the photovoltaic parameters were summarized in Table 3. The PTB7-Th:PPDI(H)-based All-PSCs exhibited a promising average PCE of 2.87%, with *V_OC_* of 0.58 V, *J_SC_* of 11.24 mA·cm^−2^, and *FF* of 0.450, and the maximum efficiency is over 3%, the highest value for A-A homopolymer-based All-PSCs. However, PBDB-T:PPDI(H)-based devices showed a much lower efficiency of 1.95%, with *V_OC_* of 0.61 V, *J_SC_* of 7.88 mA·cm^−2^, and *FF* of 0.419. In comparison to PBDB-T-based solar cells, PTB7-Th-based solar cells display lower *V_OC_* due to its high-lying HOMO energy level. However, PTB7-Th-based solar cells exhibit relatively higher *J_SC_* than PBDB-T-based solar cells, which may be partly ascribed to its better complementary absorption with PTB7-Th. The mobilities of the PTB7-Th/PPDI(H), PTB7-Th/PPDI(OD) and PTB7-Th/PNDI(OD) via the SCLC method (space charge limits current) are shown in Table S3.

**Figure 4:**
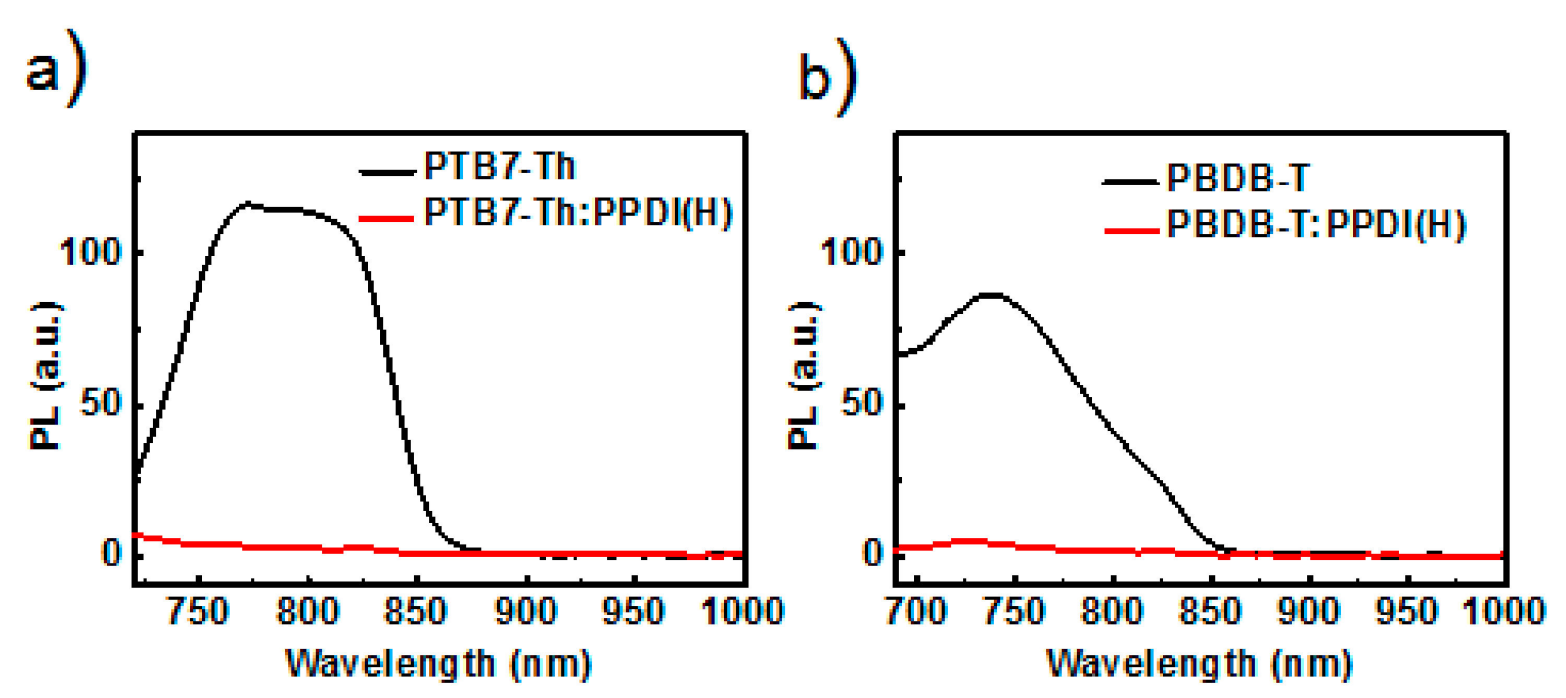
Photoluminescence spectra of PTB7-Th: PPDI(H) blend film (**a**); and PBDB-T: PPDI(H) blend film (**b**).

To understand the difference of the *J_SC_* of these two systems, absorption coefficients of the blend films were measured as shown in Figure 3b. The PBDB-T:PPDI(H) blend films possess stronger light absorption in the range of 550–650 nm due to the heavily overlapped absorption of these two materials in this area. However, the PTB7-Th:PPDI(H) blend films exhibit a much broader optical absorption in the range of 450–800 nm due to the narrow bandgap of PTB7-Th in comparison to PBDB-T:PPDI(H) blend films (450–620 nm), which may lead to generation of more excitons and higher *J_SC_*. The EQE curves of All-PSCs are shown in Figure 3c, which demonstrated that PTB7-Th-based All-PSCs possess broader photoresponse than PBDB-T-based devices, consistent with the optical absorption spectra. Importantly, PTB7-Th: PPDI(H)-based All-PSCs possess stronger photoresponse in the range from 550 to 650 nm, though the blend films absorb less optical light in this area compared to PBDB: PPDI(H) blend films, which may be ascribed to the morphology of the blend films. The exciton split was investigated by the photoluminescence of blend films as shown in Figure 4. Interestingly, the quenching efficiency of PTB7-Th: PPDI(H) blend film is 97.2%, higher than that of PBDB-T: PPDI(H) blend film (95.6%), which suggested that electron transfer from the donor polymers to the acceptor polymers in the former system is more efficient than the later one, consistent with the *J_SC_* values. Moreover, *J_SC_* was measured as a function of illumination intensity for the devices to understand the bimolecular recombination kinetics. In principle, a linear dependence of log (*J_SC_*) and log (Plight) with a slope close to 1 suggests weak bimolecular recombination in the photovoltaic devices. As shown in Figure 3d, the slope values for PTB7-Th-based and PBDB-T-based devices are 0.951 and 0.925 respectively, which revealed that the bimolecular recombination in PTB7-Th devices is less than that in PBDB-T ones, which may contribute to the larger *J_SC_*.

**Figure 5:**
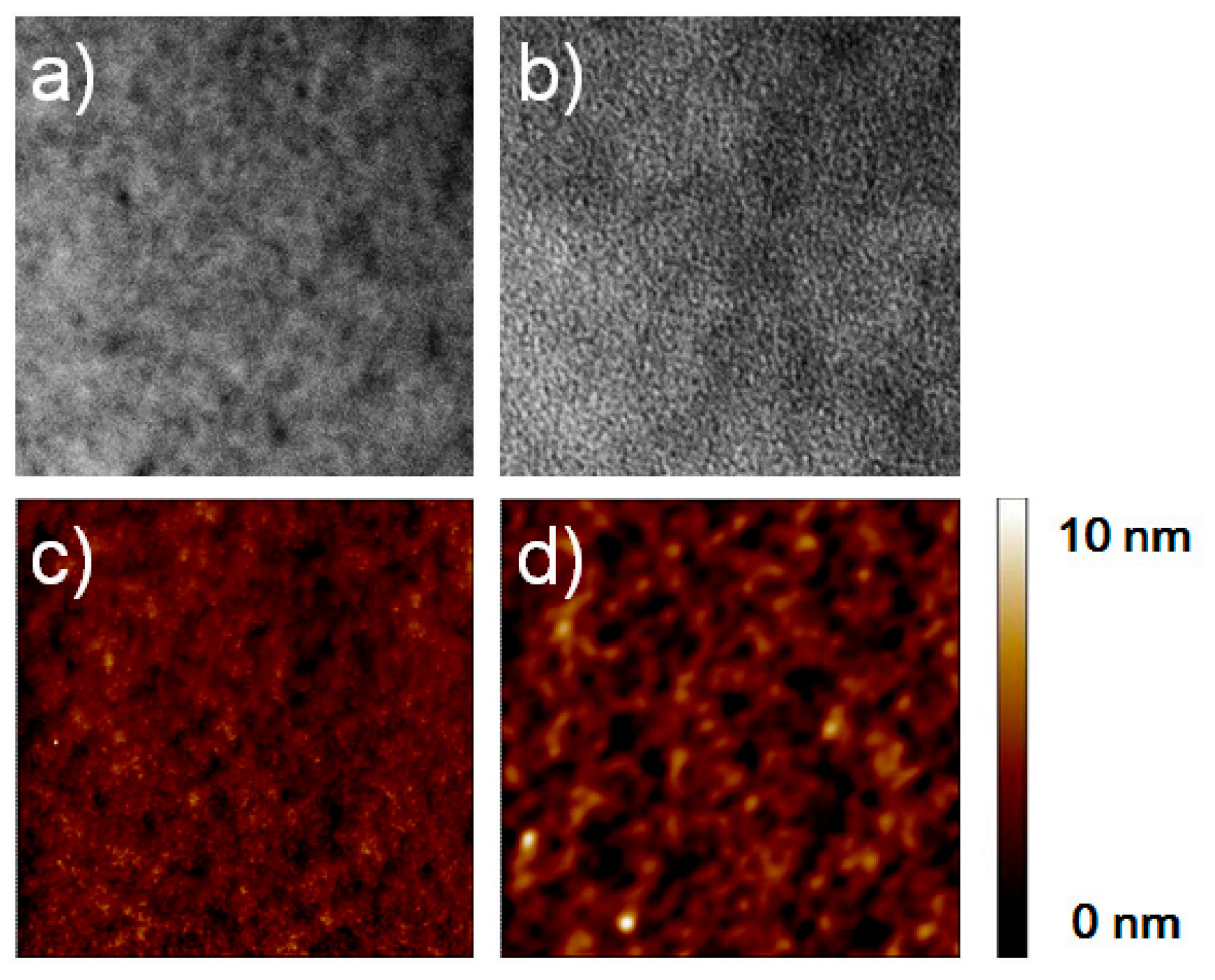
(**a**,**b**) TEM images of PTB7-Th: PPDI(H) blend film and PBDB-T: PPDI(H) blend films (the scan size is 10 μm × 10 μm); (**c**,**d**) AFM phase images of PTB7-Th: PPDI (H) blend film and PBDB-T: PPDI(H) blend films (scan size is 10 μm × 10 μm).

The morphologies of blend films are critical for photovoltaic performance, thus the PTB7-Th: PPDI(H) and PBDB-T: PPDI(H) blend films were investigated by transmission electron microscopy (TEM) and atomic force microscopy (AFM). The TEM images (Figure 5a,b) obviously show that PTB7-Th:PPDI(H) blend films exhibit featured domain size and suitable phase separation, which facilitates exciton diffusion, separation and charge transport [40]. In comparison, PBDB-T: PPDI(H) blend films demonstrate unfeatured morphologies and small domains, which are unfavorable for charge transport. On the other hand, the AFM phase images (Figure 5c,d) exhibit that the root-mean-square (RMS) roughness of PTB7-Th-based blend film and PBDB-T blend film are 0.918 nm and 1.256 nm, respectively, suggesting both films possess smooth morphologies, beneficial for the contact between blend films and interfacial layers. The powder X-ray diffraction pattern of the PPDI(H) is shown in Figure S15, which shows that no obvious diffraction peaks including crystalline or π-π stacking were observed.

## 3. Conclusions

In summary, we successfully developed Ullmann coupling polymerization as a feasible methodology to synthesize A-A type conjugated homopolymers. Upon optimizing the reaction conditions, three A-A homopolymers with respectable molecular weights were synthesized, which exhibited excellent optical, thermal and electronic properties. In combination with a narrow bandgap donor (PTB7-Th), PPDI(H)-based All-PSCs showed a promising efficiency of 3.09%, the highest value for A-A homopolymer-based OSCs. This contribution presented an important step towards development of green and cheap tools to synthesize high-performance A-A type conjugated polymers.

## Figures and Tables

**Table 1:** Optimization of the Ullmann polymerization of 2Br-PDI(H).

Entry ^a^	Solvent	*T* (°C)	Time (h)	Heat	Yield (%)	Repeat Unit ^b^
1	DMSO	80	12	Oil Bath	0	No
2	Toluene	80	12	Oil Bath	0	No
3	DMF	80	12	Oil Bath	0	No
4	DMF	150	12	Oil Bath	30	5
5	DMF	150	24	Oil Bath	32	6
6	DMF	150	5	M. W.	38	11

**^a^** All polymerization reactions used CuI as a catalyst and K_2_CO_3_ as a base; **^b^** Repeated units of products were determined by MALDI-TOF.

**Table 2:** Summarized opto-electronic properties of the polymers.

Material	λ_peak_^a^ (nm)	λ_onset_ (eV)	*E*_g_^b^ (eV)	LUMO (eV)	HOMO ^c^ (eV)
**PPDI(H)**	532	638	1.94	−4.00	−5.94
**PPDI(OD)**	545	667	1.87	−4.05	−5.92
**PNDI(OD)**	531	575	2.16	−3.84	-6.00

^a^ all data were measured in films; ^b^ *E*_g_ = 1240/λ_offset_; ^c^ HOMO = LUMO – *E*_g._

**Table 3:** Photovoltaic properties of All-PSCs under the illumination of AM 1.5 G, 100 mW·cm^2.^

Blend Film	PCE (%)	*V_OC_* (V)	*J_SC_* (mA·cm^-2^)	*FF* (%)
PTB7-Th:PPDI(H)	2.87 ± 0.22	0.58 ± 0.01	11.24 ± 0.28	45.0 ± 0.5
PBDB-T:PPDI(H)	1.95 ± 0.13	0.61 ± 0.01	7.88 ± 0.24	41.9 ± 0.4

## Supplementary Materials

The supplementary materials are available online at https://www.mdpi.com/2073-4360/11/11/1741/s1.
