# Supplementary material for: Microwave-Assisted Classic Ullmann C–C Coupling Polymerization for Acceptor-Acceptor Homopolymers
Source: Polymers (Basel). 2019 Oct 24;11(11):1741. doi: 10.3390/polym11111741 (PMC6918342; doi:10.3390/polym11111741)
Supplement: Supplementary file 1 [file polymers-11-01741-s001.pdf]

polymers

MDPI

Supplementary Material

Microwave-Assisted Classic Ullmann C-C Coupling Polymerization for Acceptor -Acceptor Homopolymers

Zijie Li, Yusheng Chen, Pan Ye, Xiangli Jia, Xiaoxi Wu, Jianfei Wu, Qinqin Shi*, Aidong Peng, and Hui Huang*.

College of Materials Science and Opto-Electronic Technology & Center of Materials Science and Optoelectronics Engineering & CAS Center for Excellence in Topological Quantum Computation & CAS Key Laboratory of Vacuum Physic, University of Chinese Academy of Sciences, Beijing 100049, P. R. China.
* E-mail: huihuang@ucas.ac.cn (H. Huang)
* E-mail: shiqinqin@ucas.ac.cn (Q. Shi)

I. Experimental section

Materials

PTB7-Th and PBDB-T were purchased from Solarmar. 2Br-PDI(H), 2Br-PDI(OD), and 2Br-NDI(OD) were synthesized according to the reproted methods [1–3].

Synthesis of PPDI(H)

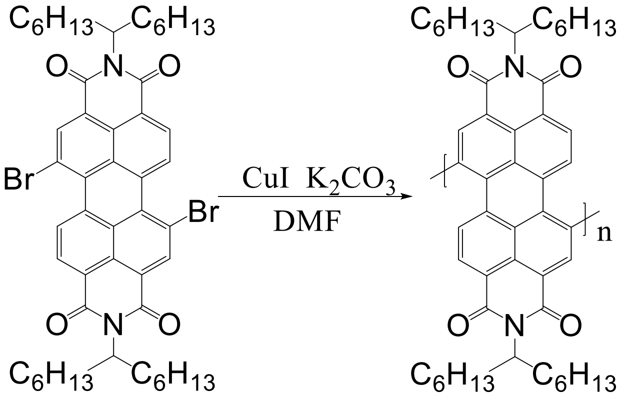

N,N′-Bis(tridecan-7-yl)-1,7-Dibromonaphtalene-3,4,9,10-Perylene Diimide (2Br-PDI(H)) (100.0 mg, 0.11 mmol) with cuprous iodide (8.4 mg, 0.044 mmol) and K_2CO_3 (30.4 mg, 0.22 mmol) were mixed in dry DMF (20 mL) under nitrogen atmosphere. After stirring at 150 °C under the microwave for 5 h, the solution slowly changed to dark purple. Then, the mixture was cooled to room temperature and filtrated to afford a dark solid. After removing the solution under vacuum, the residue was dissloved by chloroform, and washed by water and brine. Organic phase was then dried over anhydrous $MgSO_4$ and concentrated. The crude product was purified by flash chromatography on silica gel to remove high impure materials with high polar. Finally, a dark red solid (38.0 mg, yield: 38%) was obtained. ^{1}H NMR (400 MHz, chloroform-d) δ 8.80 (br, 6H), 5.04 (br, 2H), 2.3-1.3 (br, 40H), 0.71 (br, 12H). GPC (TCB, 150 °C): Mn = 7.4 k, PDI = 1.87. Calcd. for $C_{50}H_{60}N_2O_4$: C, 79.54; H, 8.28; N, 3.71. Found C, 78.73; H, 8.04; N, 3.66.

Synthesis of PPDI(OD)

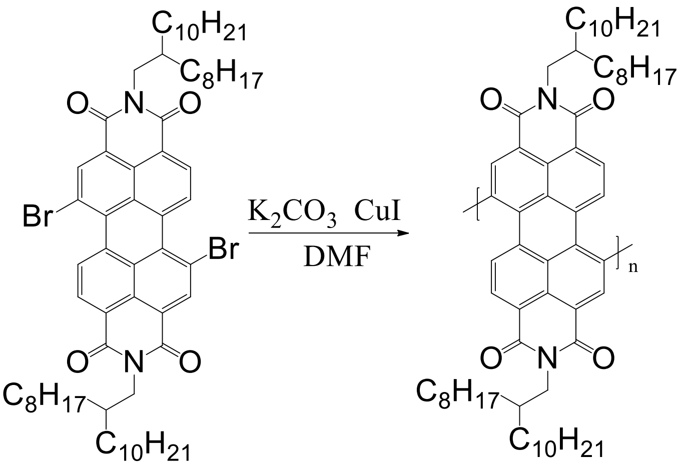

N,N′-Bis(2-Octyldodecyl)-1,7-Dibromo-3,4,9,10-Perylene Diimide (2Br-PDI(OD)) (221.8 mg, 0.20 mmol) mixed with cuprous iodide (15.2 mg, 0.08 mmol) and K_2CO_3 (55.0 mg, 0.40 mmol) were dissolved in dry DMF (4 mL) under nitrogen atmosphere. The reaction was stirring at 150 °C under microwave assistance for 5 h. The mixture was poured into water after it was cooled to room temperature. The solid was collected by filtration, which was employed for Soxhlet extraction with methanol, n-hexane and tetrahydrofuran respectively. The tetrahydrofuran portion was then concentrated and poured into methanol. The precipitates were collected by filtration and dried in air to afford 100 mg (yield 52.5%) a dark-puple solid. ^{1}H NMR (400 MHz, chloroform-d) δ 8.54 (br, 6H), 4.08 (br, 4H), 2.15-1.60 (br, 66H), 0.95 (br, 12H). GPC (TCB, 150 °C): Mn = 12.5 k, PDI = 1.84. Calcd. for $C_{64}H_{88}N_2O_4$: C, 80.96; H, 9.34; N, 2.95. Found C, 80.07; H, 8.93; N, 2.87.

Synthesis of PNDI(OD)

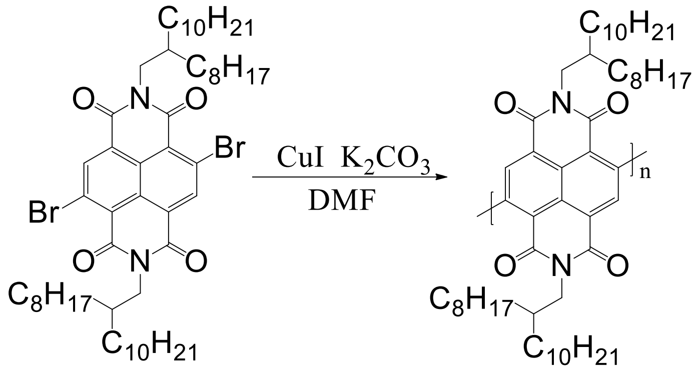

N,N′-Bis(2-octyldodecyl)-2,6-dibromonaphtalene-1,4,5,8-tetracarboxylic acid bismide (2Br-NDI(OD)) (197.0 mg, 0.20 mmol) mixed with cuprous iodide (15.2 mg, 0.08 mmol) and K_2CO_3 (55.0 mg, 0.40 mmol) were dissolved in dry DMF (20 mL) under nitrogen atmosphere. The reaction was stirring at 150 °C under microwave for 5 h. Afterwards, the mixture was cooled down to room temperature and poured into water. The solid was collected by filtration, followed by soxhlet extraction with methanol and n-hexane. The n-hexane portion was then concentrated and poured into methanol. The precipitates were collected and dried in the air to afford 100 mg (yield 60.4%) a black-red solid. ^{1}H NMR (400 MHz, chloroform-d) δ 8.50 (br, 2H), 4.18 (br, 4H), 1.25 (br, 66H), 0.98 (br, 12H). GPC (TCB, 150 °C): Mn = 5.9 k, PDI = 1.86. Calcd. for $C_{54}H_{84}N_2O_4$: C, 78.59; H, 10.26; N, 3.39. Found C, 78.73; H, 9.67; N, 3.01.

Table S1. The Results of Ullmann C-C Coupling Polymerizations.

Product	Yield	Mn	PDI
**PPDI(H)**	38.0%	7.4k	1.87
**PPDI(OD)**	52.5%	12.5k	1.84
**PNDI(OD)**	60.4%	5.9k	1.86

Device Fabrication

Sandwich-structure inverted solar cells consisted of a stack of ITO-coated glass/ZnO/active layer/MoO_3/Ag. ITO-coated glass substrates were first cleaned by ultrasonic agitation in detergent, deionized water, acetone, and isopropanol in sequence, followed by drying at 80 °C in an oven overnight and then UV ozone treatment for 30 min. 25 μL of ZnO precursor (zinc acetate dihydrate) solution was spin-coated on top of the pre-cleaned ITO (4500 rpm, 40 s). After coating, the films were heated at 200 °C for 30 min.

Afterward, a chlorobenzen solution containing 10 mg/mL PTB7-Th, 13 mg/mL acceptor and 3% 1-chloronaphthalene as additive or containing 10 mg/mL PBDB-T, 10 mg/mL acceptor and 1% 1,8-diiodooctane as additive was spin-coated on the substrate to afford an active layer inside of the glove box. Then, the thin films were transferred into a vacuum evaporator connected to the glove box. MoO_3 (10 nm) and Ag (100 nm) were deposited sequentially by thermal evaporation at a pressure under 10^{-5} Pa.

Device Characterization

The current density-voltage (*J-V*) characterizations were scanned at 10 mV intervals under air mass (AM) 1.5G using a Newport solar simulator. The average results were obtained from 15 devices. The input photon to converted current efficiency (IPCE) was measured by Newport IPCE system.

General Measurements

The molecular weight of the polymers was measured by Angilent Technologies PL-GPC 220 High Temperature Chromatograph. ^1H NMR were obtained on a JNM-ECZ 400 MHz nuclear magnetic resonance spectrometer and calibrated against the peak of tetrame-thylsilane (TMS, 0 ppm). MALDI-TOF MS spectra were obtained on a Bruker Daltionics Biflex III MALDI-TOF Analyzer. Element analysis (EA) was measured on a Flash EA 1112 elemental analyzer. Thermogravimetric analyzer (TGA) were tested on a Q50 at a heating rate of 10°C·min^{-1} under a nitrogen atmosphere. Differential scanning calorimeter (DSC) was tested by Q2000 at a heating rate of 10°C·min^{-1} under a nitrogen atmosphere. Density functional theory (DFT) calculations were calculated on the B3LYP/6-31G(d,p) level. Charge distribution of the molecules was calculated by Mulliken population analysis. All torsional potentials were computed at 10º intervals. For each data point, we maintained the dihedral angle and optimized geometry on all remaining degrees of freedom. And all small molecules simplified side chains with methyl. UV-vis absorption spectra were tested on Gary 60 UV-vis Spectrophotometer. Photoluminescience (PL) spectra were tested by Cary Eclipse Fluorescence Spectrophotometer, all films samples were spin-casted on quartz glass substrates, and the film thickness was obtained by DektakXT. Cyclic voltammetry (CV) measurements were proceeded on a CHI600E electrochemical workstation. The glassy-carbon disk electrode (working electrode) coated with the ORCN and ERCN films were mearsured with a potential scan rate of 20mV/s in 0.1 M tetrabutylammonium hexafluorophosphate (Bu_4NPF_6) acetonitrile solution. The platinum wire and Ag/AgCl electrode were used as the counter electrode and the reference electrode. The ferrocene/ferrocenium (Fc/Fc$^+$) was used as external standard. The HOMO/LUMO levels were calculated based on the equations $E_{HOMO}/E_{LUMO} = -e\ (\varphi_{oxd}/\varphi_{red} + 4.80 - \varphi_{Fc/Fc+})$ (eV), where φ_{oxd} and φ_{red} were obtained from the onset of oxidation and reduction. The $\varphi_{Fc/Fc+}$ was obtained from the half-wave potential of ferrocene. All film samples of AFM were spin-coated using the same conditions for OSCs on ITO glass substrates with a ZnO interlayer. AFM images were obtained by NTEGRA Prima in the tapping mode. TEM images were performed by HT7700Ex instrument at 110 kV accelerating voltage.

All film samples of TEM images were prepared by two steps. Firstly, the active layers were spin-casted using the same conditions for solar cells on ITO glass substrates with a PEDOT:PSS interlayer, then the active layers were floated by dissolving the water soluble PEDOT:PSS on the surface of demineralized water and picked up with hexagonal-mesh copper TEM grids.

Ⅱ. Supporting figures.

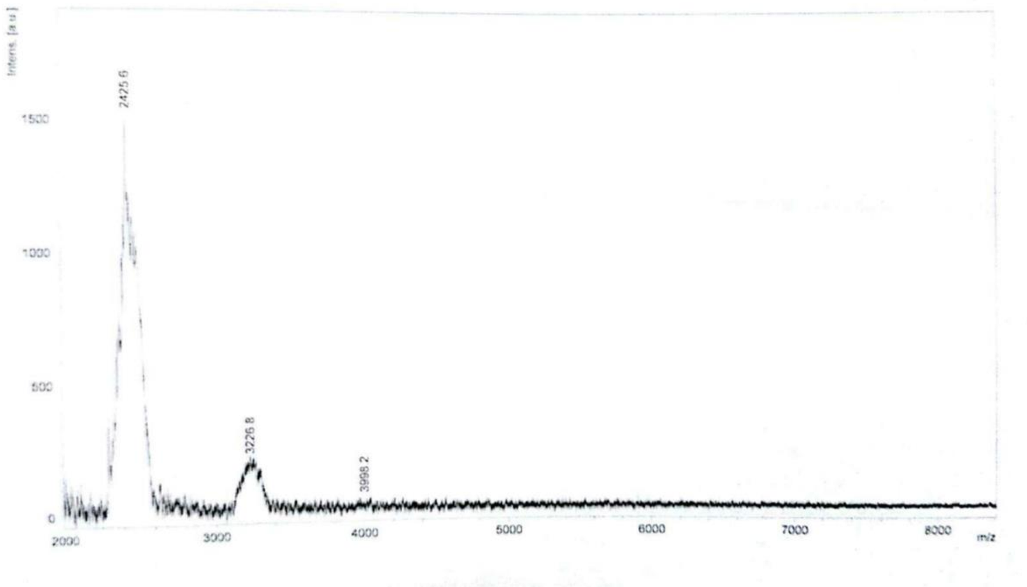

Figure S1. MALDI-TOF of Entry 4.

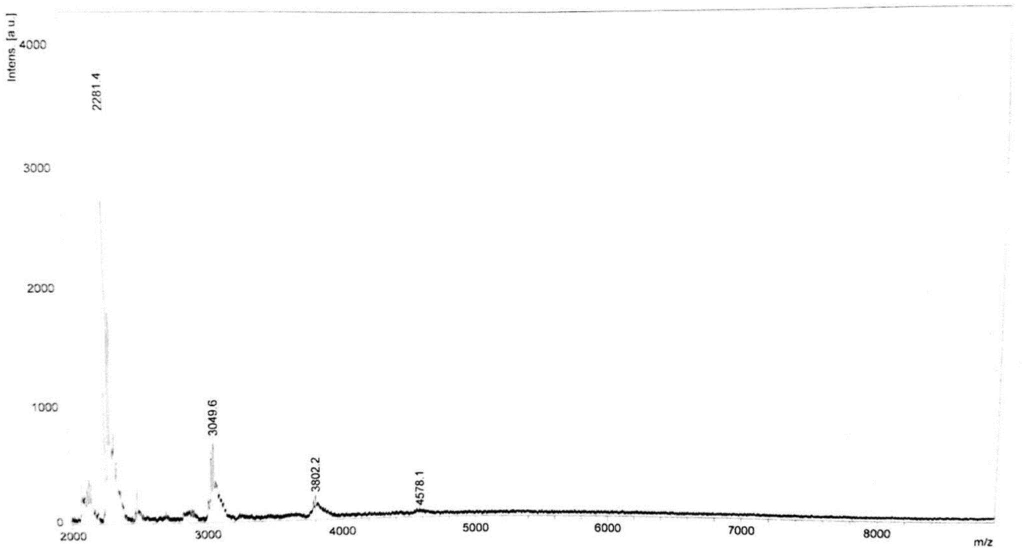

Figure S2. MALDI-TOF of Entry 5.

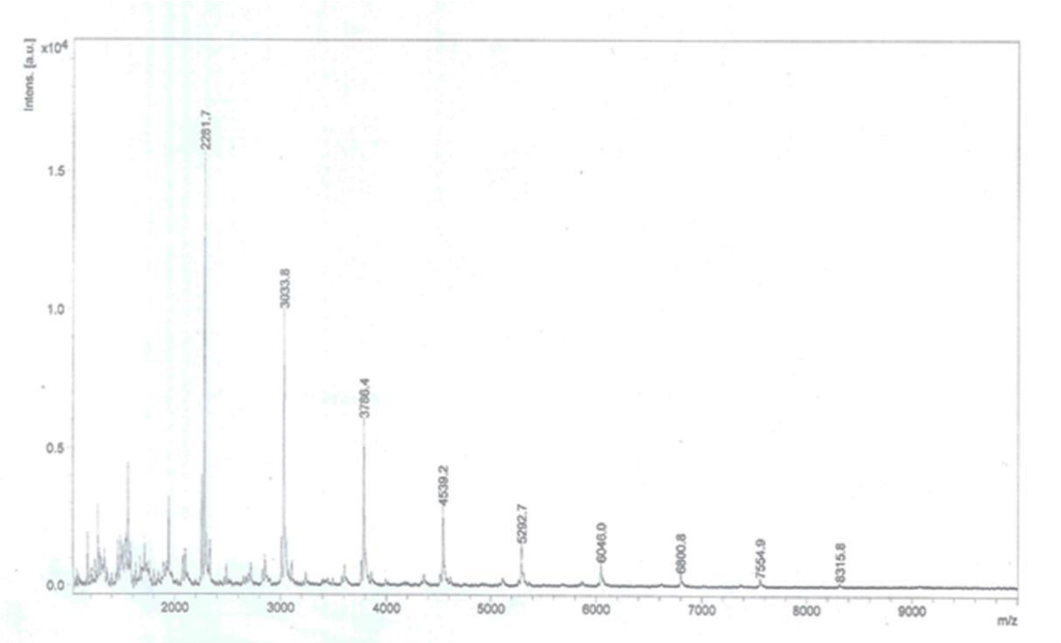

Figure S3. MALDI-TOF of Entry 6.

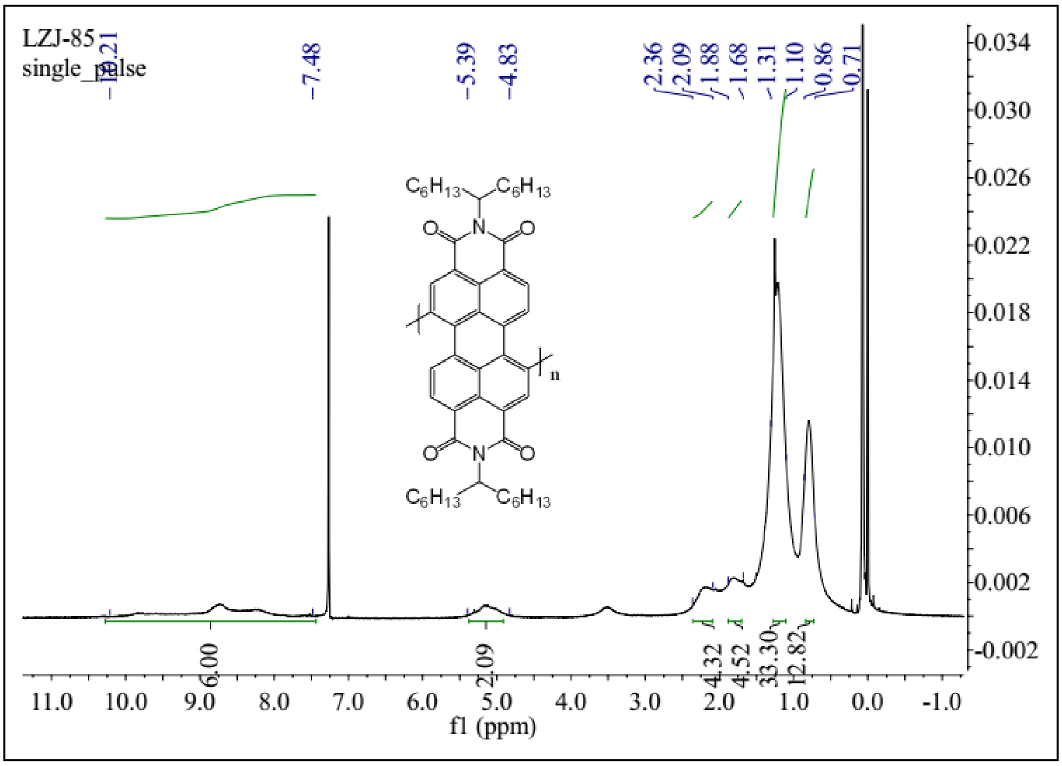

Figure S4. ^{1}H NMR of PPDI(H).

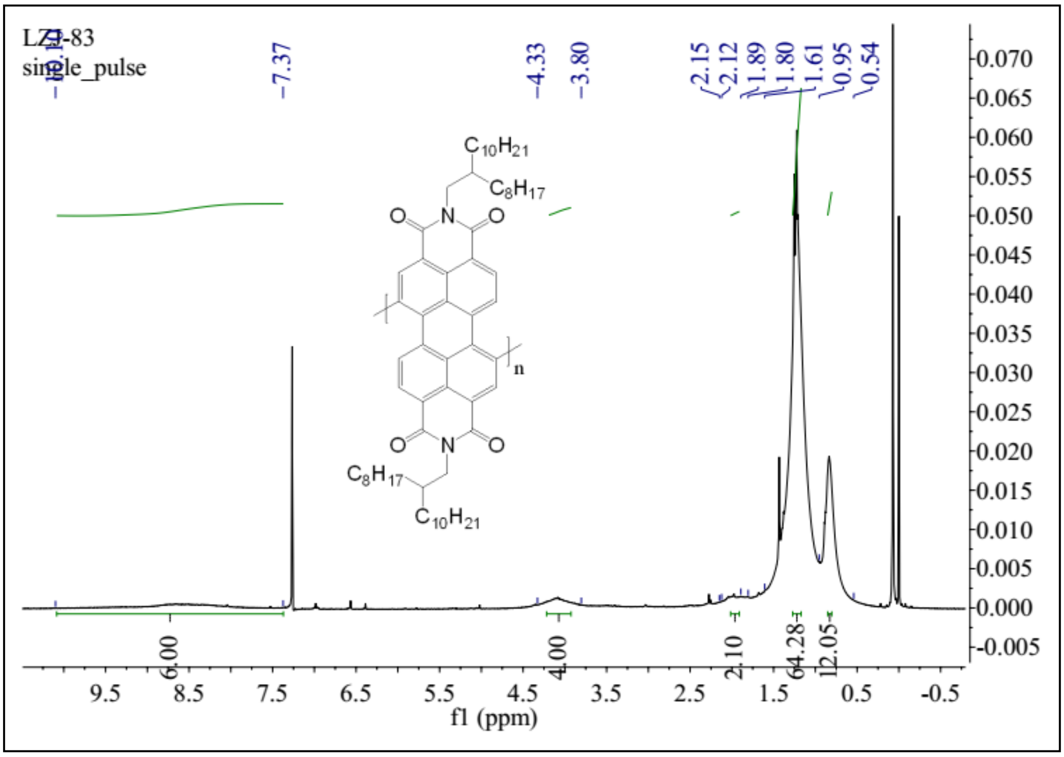

Figure S5. ^{1}H NMR of PPDI(OD).

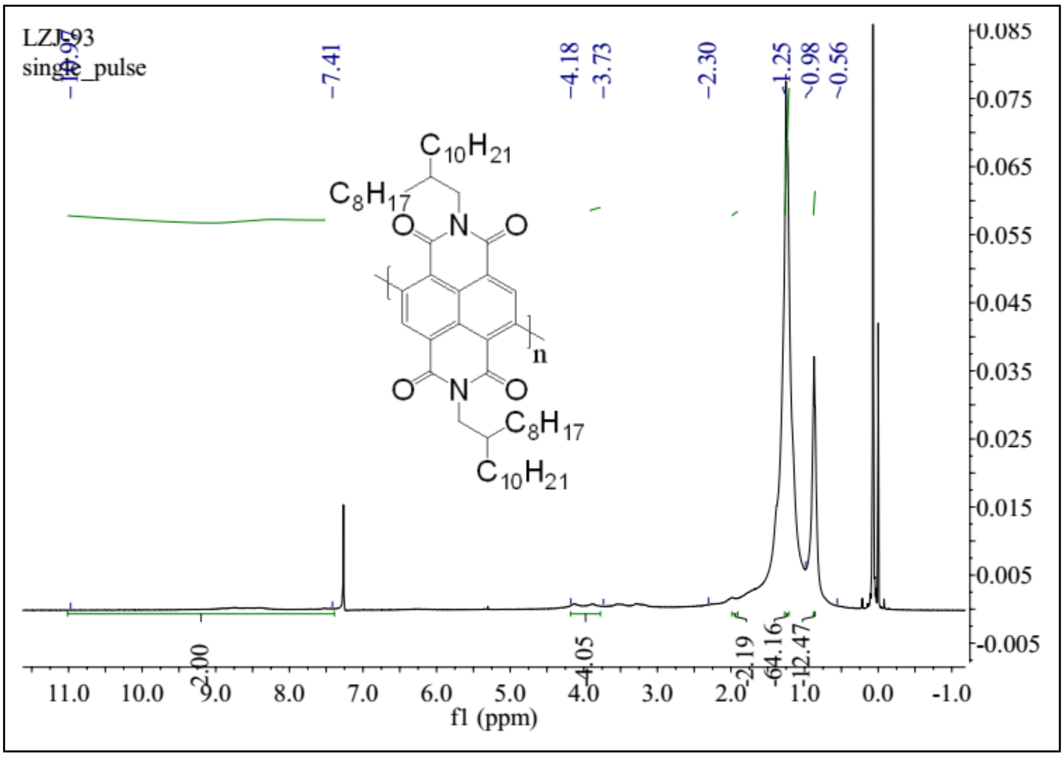

Figure S6. ^{1}H NMR of PPDI(OD).

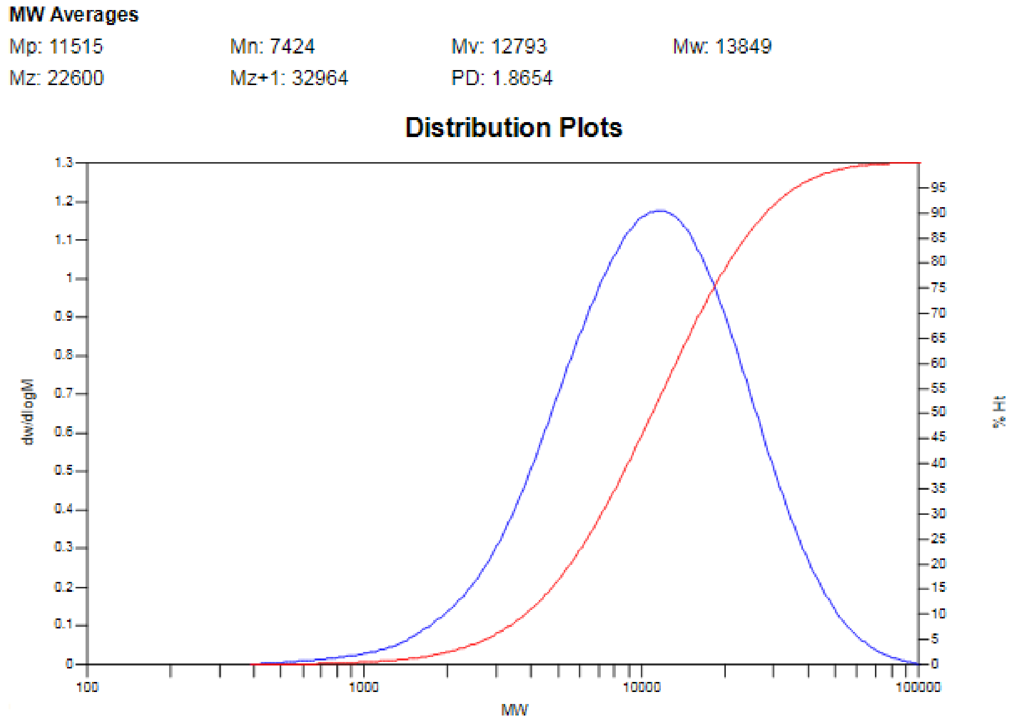

Figure S7. GPC of the PPDI(H).

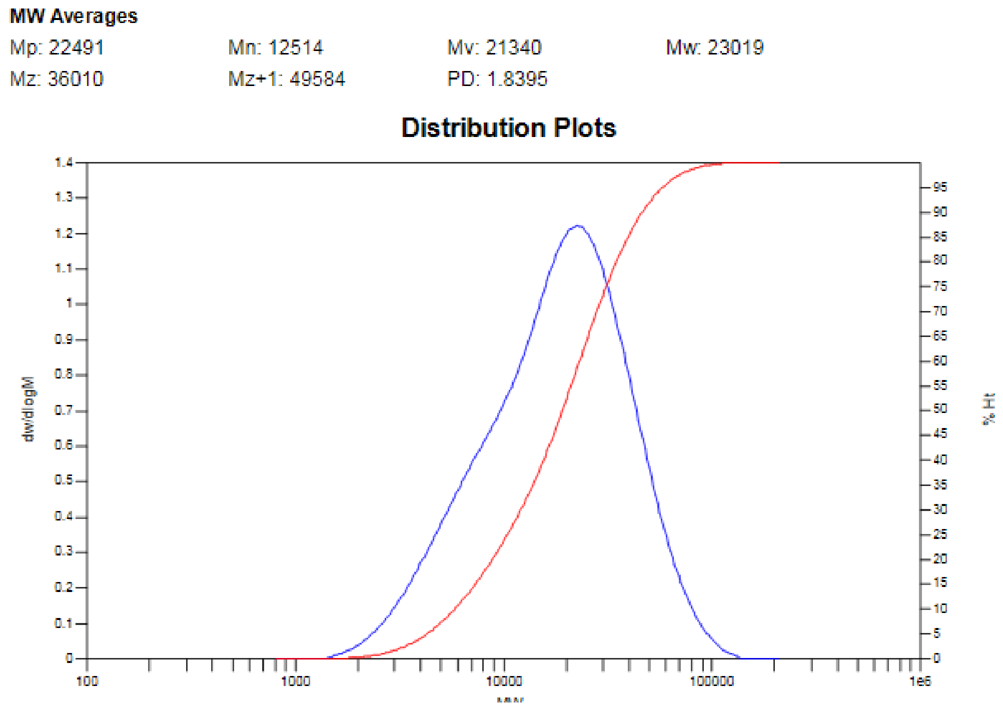

Figure S8. GPC of the PPDI(OD).

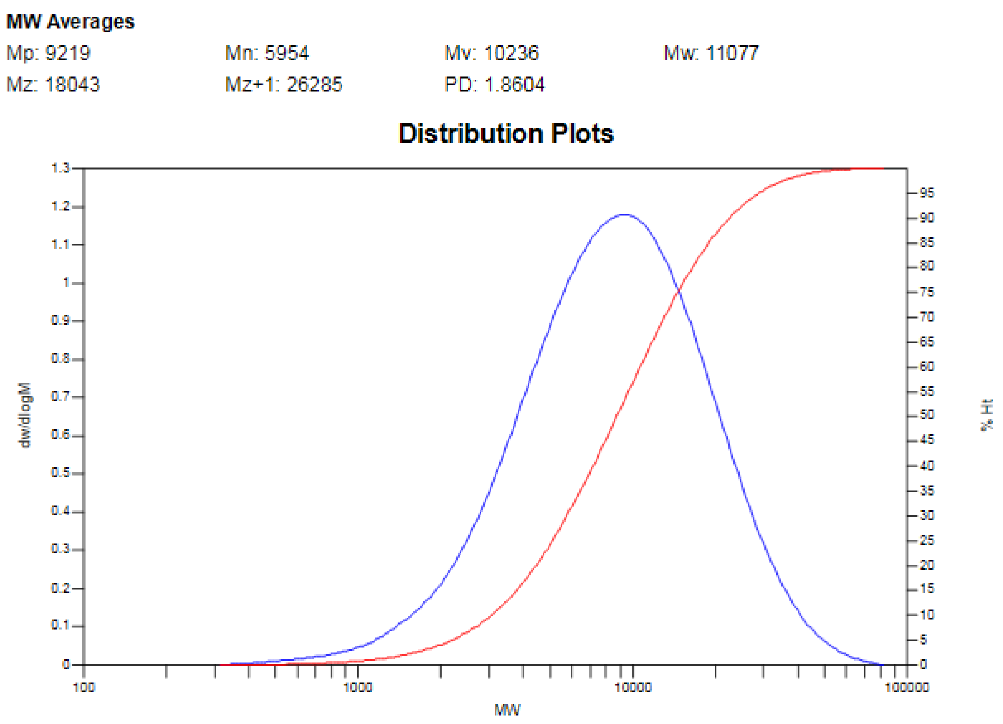

Figure S9. GPC of the PNDI(OD).

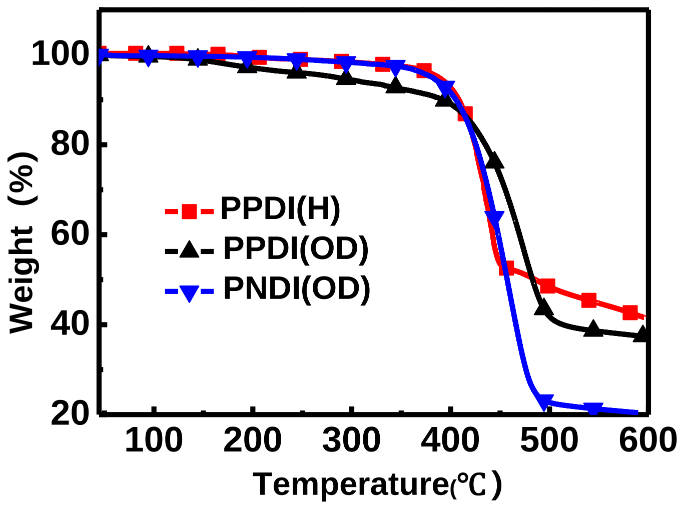

Figure S10. TGA measurement of PPDI(H), PPDI(OD), and PNDI(OD).

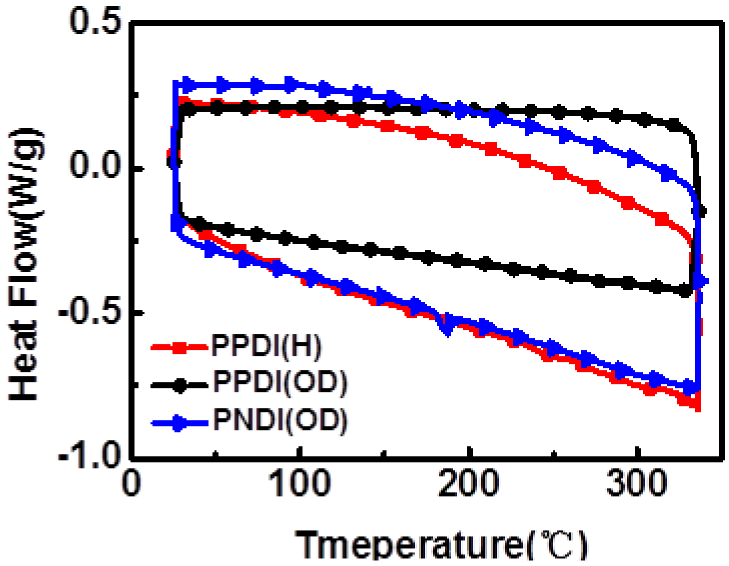

Figure S11. DSC thermogram of all PPDI(H), PPDI(OD) and PNDI(OD).

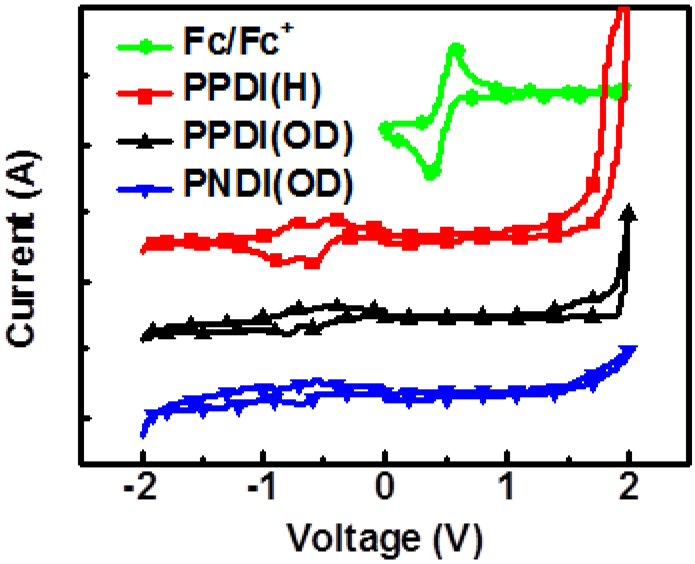

Figure S12. Cyclic voltammograms of PPDI(H), PPDI(OD), and PNDI(OD).

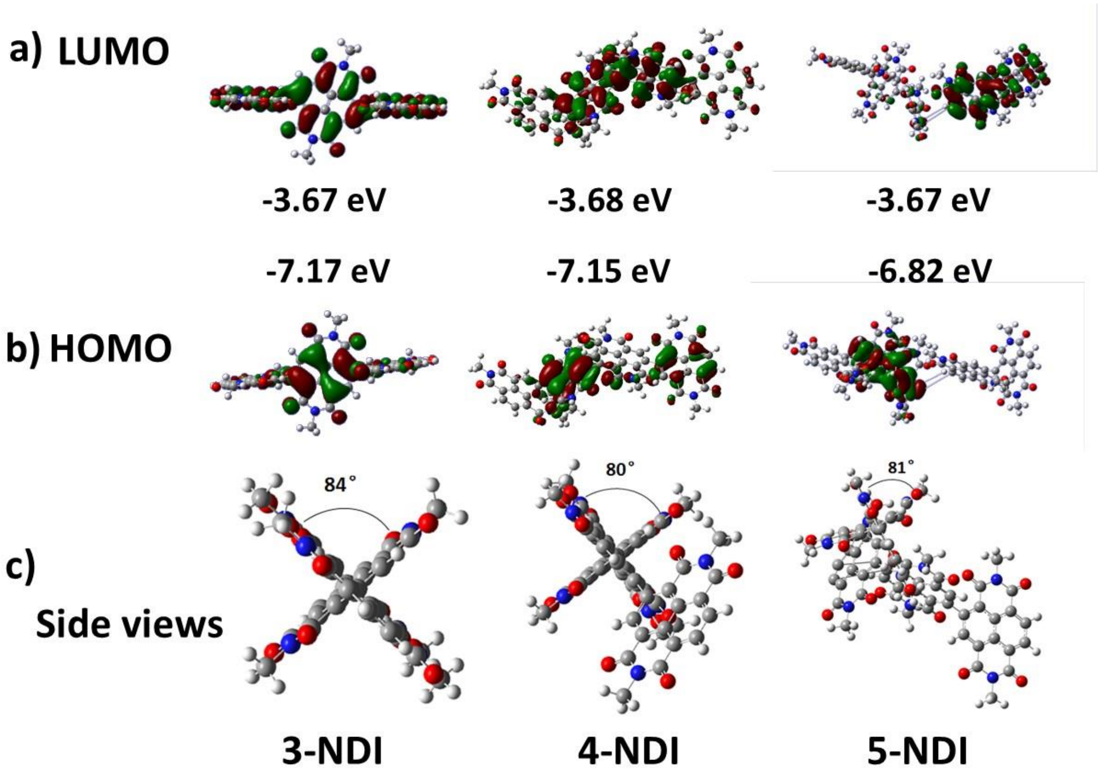

Figure S13. a), b) Optimized geometry, calculated HOMO and LUMO orbitals of the 3-NDI, 4-NDI and 5-NDI. **c)** Optimized geometry of three molecules shown from side view.

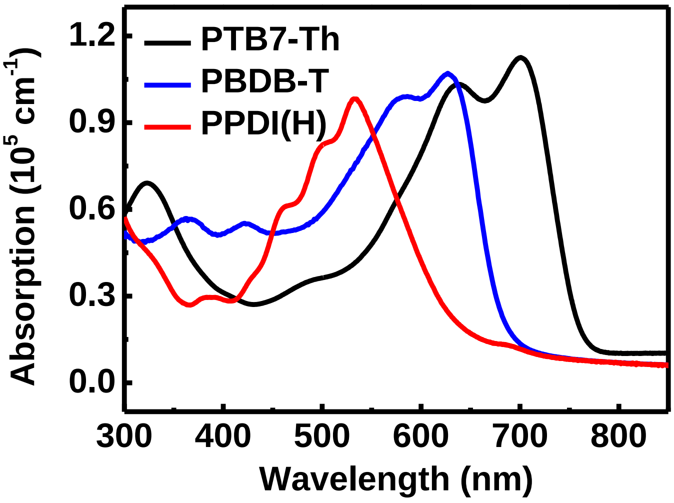

Figure S14. Absorption coefficenct of PTB7-Th, PBDB-T and PPDI(H).

Table S2. Energy levels of all PPDI polymers.

Product	3-PDI	4-PDI	5-PDI
**LUMO(eV)**	-3.68	-3.71	-3.73
**HOMO(eV)**	-5.95	-5.96	-5.96
**Bandgap**	2.27	2.25	2.23

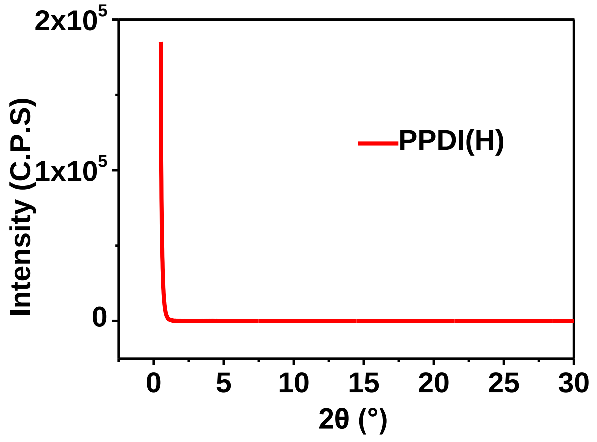

Figure S15. The powder X-ray diffraction (XRD) scans of PPDI(H).

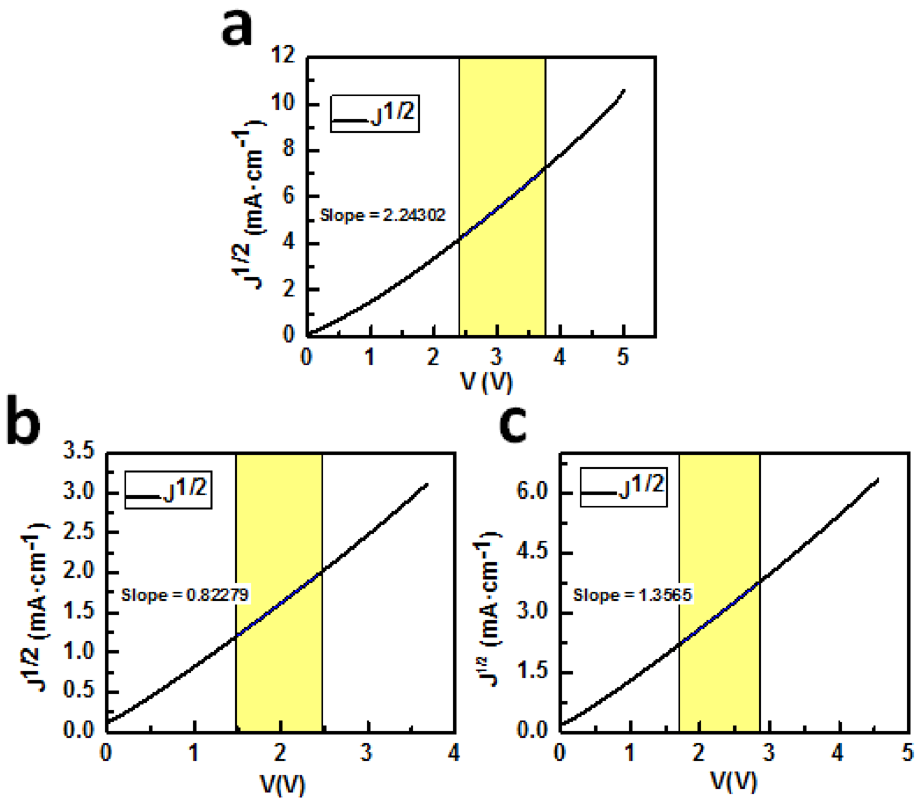

Figure S16. The mobility curve of the PTB7-Th/PPDI(H) **a)**, PTB7-Th/PPDI(OD) **b)** and PTB7-Th/PNDI(OD) **c)** via SCLC method.

Table S3. The mobility data of the PTB7-Th/PPDI(H) a), PTB7-Th/PPDI(OD) b) and PTB7-Th/PNDI(OD) c) via SCLC method.

**PTB7-Th/PPDI(H)**	**PTB7-Th/PPDI(OD)**	**PTB7-Th/PNDI(OD)**
4.00×10^{-5} cm^2 V^{-1} s^{-1}	0.54×10^{-5} cm^2 V^{-1} s^{-1}	1.48×10^{-5} cm^2 V^{-1} s^{-1}

1. Chen, Y.; Ye, P.; Zhu, Z. G.; Wang, X.; Yang, L.; Xu, X.; Wu, X.; Dong, T.; Zhang, H.; Hou, J.; Liu, F.; Huang, H., *Adv. Mater.* **2017,** 29,1603145.
2. Chen, Z.; Zheng, Y.; Yan, H.; Facchtti, A., *J. Am. Chem. Soc.* **2009,** 131, 8-9.
3. Li, L.; Hadt, R. G.; Yao, S.; Lo, W. Y.; Cai, Z.; Wu, Q.; Pandit, B.; Chen, L. X.; Yu, L., *Chem. Mater.* **2016,** 28, 5394-5399.

Ⅲ.Appendix:

For Table of Contents use only：

Microwave-assisted Classic Ullmann C-C Coupling Polymerization for A-A Homopolymers

Zijie Li, Yusheng Chen, Pan Ye, Xiangli Jia, Xiaoxi Wu, Jianfei Wu, Qinqin Shi*, Aidong Peng, and Hui Huang*.

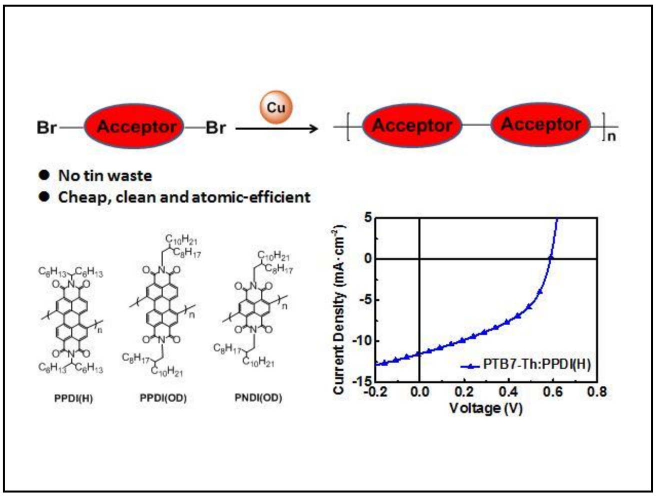

Ullmann coupling polymerization was developed as a universal methodology to synthesize a series of PDI-based A-A type conjugated homopolymers. In combination with a narrow bandgap donor (PTB7-Th), PPDI(H) based all-PSCs showed a promising efficiency of 3.09%, the highest value for A-A homopolymer-based OSCs. This contribution presented an important step to develop green and cheap methodologies to synthesize high-performance conjugated polymers.
